# A review on the physicochemical and biological applications of biosurfactants in biotechnology and pharmaceuticals

**DOI:** 10.1016/j.heliyon.2022.e10149

**Published:** 2022-08-08

**Authors:** Vikrant Abbot, Diwakar Paliwal, Anuradha Sharma, Poonam Sharma

**Affiliations:** a Department of Biotechnology and Bioinformatics, Jaypee University of Information Technology, Waknaghat, Solan (Himachal Pradesh) 173234, India; b Faculty of Pharmaceutical Sciences, PCTE Group of Institutes, Campus-2, Near Baddowal Cantt. Ferozpur Road, Ludhiana (Punjab) 142021, India

**Keywords:** Biosurfactants, Microbes, Applications, Biotechnology, Pharmaceuticals

## Abstract

Biosurfactants are the chemical compounds that are obtained from various micro-organisms and possess the ability to decrease the interfacial tension between two similar or different phases. The importance of biosurfactants in cosmetics, pharmaceuticals, biotechnology, agriculture, food and oil industries has made them an interesting choice in various physico-chemical and biological applications. With the aim of representing different properties of biosurfactants, this review article is focused on emphasizing their applications in various industries summarizing their importance in each field. Along with this, the production of recently developed chemically and biologically important biosurfactants has been outlined. The advantages of biosurfactants over the chemical surfactants have also been discussed with emphasis on the latest findings and research performed worldwide. Moreover, the chemical and physical properties of different biosurfactants have been presented and different characterization techniques have been discussed. Overall, the review article covers the latest developments in biosurfactants along with their physico-chemical properties and applications in different fields, especially in pharmaceuticals and biotechnology.

## 1 Introduction

Surfactants or surface-active agents are the chemical compounds containing both hydrophobic as well as hydrophilic regions making them amphiphilic in nature. They alter the properties of the surface or interfacial tension of a fluid that allow forming a microemulsion, which lead to solubilization of colloidal solutions, micellar systems and water-oil suspensions [1, 2]. This phenomenon of surfactants makes them an essential component in many sectors such as pharmaceuticals, cosmetics, agriculture, oils and food industries etc. Surface tension, which measures the force of attraction between the liquid-liquid, liquid-solid, or liquid-gas interfaces that are in contact with it, is the elastic tendency of liquid surfaces [3, 4]. Small liquid drops and soap bubbles with a roughly spherical shape are the most common manifestations of this phenomena. It is a critical parameter for evaluating the effectiveness of a surfactant, leading to decrease in the surface tension when their concentration in the solution increases, which results in micelle formation [5]. Micelle formation occurs due to the inability of lipophilic region of the surfactant to form hydrogen-bond in aqueous phase and thereby contributing to the system’s free energy. Dealing with this increased free energy is required to aid in isolation of hydro-carbon tail from water by its absorption onto organic or aqueous surfaces enabling the hydrophobic region oriented towards the centre while hydrophilic region facing water [6, 7, 8]. Surfactants decrease the interfacial tension between two molecules in a liquid by decreasing the intermolecular interactions between them. The surfactants consist of large hydrocarbon chains in their structure. When these surfactants are incorporated in a liquid solvent system, they occupy the intermolecular spaces between the liquid particles. This result in weak intermolecular forces between the molecules and result in decreased interfacial tension. The decrease in surface tension between the molecules allows the solid or liquid solute particles to form hydrophilic or hydrophobic interactions with the solvent making immiscible fluids miscible, due to formation of new additional surfaces [9]. Micelles are defined as aggregated molecules containing both hydrophobic and hydrophilic groups, with hydrophilic groups oriented towards water, while hydrophobic groups oriented towards oil [10, 11]. The formation of micelles leads to distribution of hydrophobic compounds into the pseudocore residing in the centre thereby enhancing solubility. This leads to better dispersion of compounds and leads to increased mobility of adsorbed hydrophobic soil contaminants by decreasing the capillary forces. The minimum surfactant concentration at which solution properties of a liquid shows an abrupt change is regarded as Critical Micelle Concentration (CMC) [12].

**Figure 1:**
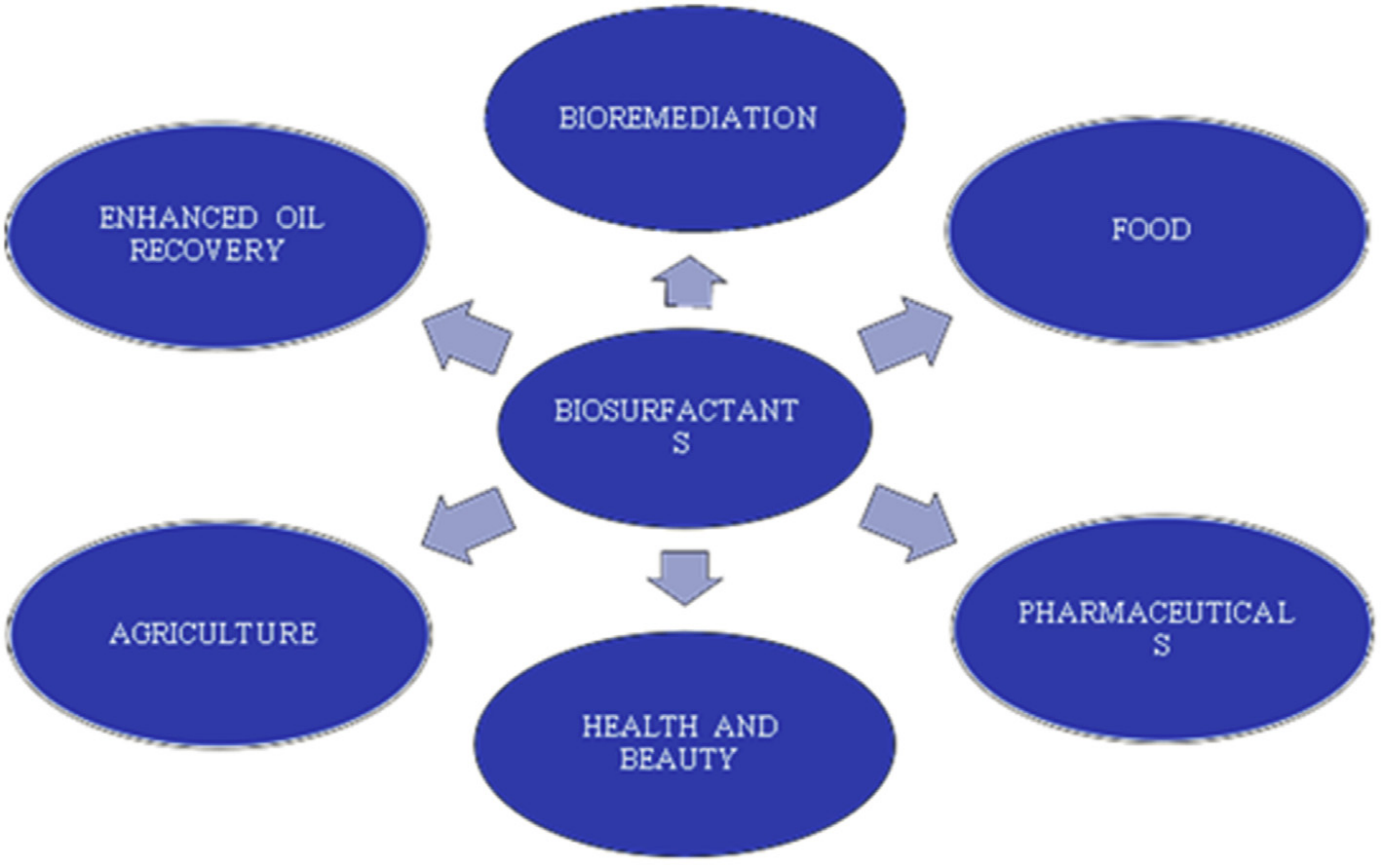
Applications of biosurfactants in various industries.

Biosurfactants are biological compounds of microbial origin that have surface active properties and can be used effectively instead of chemical surfactants [13]. They have lower CMC compared to that of synthetic ones and they lead to higher decrease in surface tension of oil-water or air-water interfaces at a very low concentration [14, 15]. These characteristics make biosurfactants a good choice for froth stabilization and emulsification. Microbial surfactants provides more efficiency and advantages over the chemical ones, in particular with respect to their biodegradability, mild creation conditions, ecological compatibilities, lower toxicity, higher selectivity, explicit action over extraordinary temperatures, pH and salinities, thus promoting their use in various industries as represented in Figure 1 [16, 17, 18].

## 2 Biosurfactants

Amphiphilic substances with hydrophilic tail and hydrophobic head ends are termed as biosurfactants. Cyclic peptides, phosphates, carboxyl acids, or alcoholic groups are typically found in the hydrophilic region of biomolecules, whereas long chain fatty acids, hydroxyl fatty acids, α-alkyl-β-hydroxyl fatty acids, etc. are found in the hydrophobic zone [19]. These moieties can likewise incredibly affect the interfacial conduct and mass exchange, other than bringing down the fluids surface pressure, as they likewise bring down the interfacial strain between diverse fluid stages on the limit of the interface that exists between stages that are immiscible [20, 21]. Microbial surfactants possess an intense property to decrease the tension between surfaces and show diverse properties such as emulsification, lubrication ability, phase dispersion, detergency etc. They are majorly used in food, petroleum, cosmetics industries, bioremediation, environmental and pharmaceutical industries [22, 23]. Surfactant molecules are amphipathic in nature that get partitioned between two phases, having varying degrees of polarity. At their micelle concentration, these microbial surfactants works best and this value can be around 10 to 40 times lower than the chemical surfactants [24]. Due to moisturizing and low toxicity properties of complex lipids such as lipopeptides and glycolipids, microbial surfactants are commonly considered as low or non-poisonous compounds. Biosurfactants segment at interfaces with particular polarities along with hydrogen holding, thus influencing the attachment of microorganisms [25]. The presence of biosurfactant in a media can be detected by various techniques using colorimetric analysis, emulsification index determination, drop collapsing test and thin layer chromatography [26, 27].

## 3 Production

Biosurfactants are produced from non-exhaustible renewable sources thus having high surface activities, along with high specificity and the ability to function under extreme conditions [28]. They are generally produced by aerobically growing microorganisms utilizing feedstock’s as source of carbohydrates, fats etc. Microbial surfactants are actively secreted in the culture media and are supposed to aid in the growth of microbes by facilitating transport of solutes that are insoluble across the cell membrane [29, 30]. They are usually non-ionic or anionic moieties and have both lipophilic as well as hydrophilic groups.

Both high and low molecular weight biosurfactants are developed from microorganisms. Low molecular weight biosurfactants incorporate glycolipids like trehalose, fructose lipids and sophorolipids while peptidyl lipids incorporate surfactin, polymyxin, and so forth. Surfactin is a biosurfactant produced by *Bacillus subtilis*, which possesses amphiphilic structure and serves as a potent inhibitor of bacterial and tumour growth, and are associated in decreasing the surface tension of the fluids [31, 32, 33]. On the other hand, biosurfactants with higher molecular weights are amphiphilic polysaccharides that provide formation of stable emulsions and do not lower the interfacial tension [34]. Due to their capacity of forming strong emulsions, they facilitate in sturdy adhesion of bacteria to hydrophobic surfaces. Most of the biosurfactants are obtained from *Arthrobacter, Bacillus subtilis, Pseudomonas aeruginosa, Acinetobacter calcoaceticus, Candida lipolytica, Corynebacterium, Nocardia, Bacillus subtilis,Bacillus licheniformis, Candida* spp. and many others [35, 36, 37]. Majority of biosurfactants are comprised of substrates which are insoluble in water. Some researchers have acquired biosurfactants with the aid of developing a culture of *Candida lipolytica* on groundnut oil. Similarly, *Candida glabrata* grown on vegetable oil also provide biosurfactants. Few studies also describes production of rhamnolipids from low value carbon resources from *Pseudomonas aeruginosa* [38, 39, 40, 41].

Rhamnolipids have been acquired by using restaurant waste oil in bioreactor configuration from strains of *Pseudomonas aeruginosa* [42]*.* Siddhartha *et al.* reported another low-cost medium having cassava waste water and waste cooking oil to obtain rhamnolipids [43]. Endophytic fungi have also been used to attain biosurfactants, they are found in tropical regions on trees like *Piper hispidum*, which act as potent source for ultimate production of biosurfactants as reported by Silva *et al.* [44]. Many researchers have demonstrated the importance of glycolipids and lipopeptides in producing microbial biosurfactants. Some of the certain progresses made in this research includes production of rhamnolipids from *Pseudomonas aeruginosa* [45], trehalolipids created by *Rhodococcus erythropolis* [46], sophorolipids produced from *Candida bombicola* [47] and mannosyl erythritol lipids (MELs) created by *Pseudozyma* yeasts [48]. A list of some common biosurfactants along with the microorganisms from which they have been produced is presented in Table 1.

**Table 1:** Some common biosurfactants obtained from various microorganisms.

S. No.	Biosurfactants	Class	Micro-organism	References
1	Rhamnolipid	Glycolipids	*Pseudomonas aeruginosa*, other species of *Pseudomonas*	[49]
2	Surfactin	Lipopeptide	*Bacillus subtilis*	[14]
3	Emulsan	Polymer	*Acinetobacter calcoaceticus*	[50]
4	Sophorolipids	Glycolipids	*Candida bombicola*	[51]
5	Liposan	Polymer	*Candida lipolytica*	[52]
6	Viscosin	Lipoproteins	*Pseudomonas fluorescens*	[53]
7	Alasan	Polymer	*Acinetobacter radioresistens*	[54]
8	Trehalolipids	Glycolipids	*Rhodococcus* sp*.*	[55]
9	Arthrofactin	Polypeptide	*Arthrobacter* spp*.*	[56]
10	Lipomannan	Polymer	*Candida tropicalis*	[57]

For upgrading the conditions to enhance the biosurfactant production, the metabolic pathway of the producing organism as well as the chemical structure of biosurfactants should be well known. By employing this information, *Zouari et al.* utilized poultry farming waste and buttermilk as potential sources of nitrogen and carbon [58]. This mixture was found to be proficient for creation of SPB1 lipopeptide. These days, oil determined manufactured surfactants end up being the most broadly utilized across various ventures, essentially because of significant expense of creation of biosurfactants, additionally the way that wide scale creation of these has not been characterized at this point to satisfy the modern needs. This area is being worked upon to track down ways of reducing the cost of creation, mainly focusing on use of alternate substrates, microbial culture and culture conditions. The utilization of minimal expense media can likewise help in reducing the cost of biosurfactants production [59, 60, 61].

A huge amount of organic waste is being produced via food, forest, municipalities and agriculture industries. Despite the fact that biosurfactants are very beneficial over engineered surfactants, they actually are bit costly to prepare. The best way to lessen their expense of production is through modification in fermentation technologies and using renewable substrates and different microbial strains [62, 63]. Further optimizing operating parameters like temperature, pH, agitation speed, duration and recovery of products make them more efficient, but still a bit costly compared to synthetic ones. Use of low cost agro-industrial waste may overcome the issue of cost [64]. It is necessary to develop techniques which allow easy production and downstream processing of the product so as to be able to be used at a large scale. It relies on the design, organization and centralization of surfactant to pick a feasible recuperation strategy. Xiaowei *et al.* solved this problem by using industrial by-products like vegetal waste oil and glycerol in appropriate concentrations. It was observed that these by-products did not restrained development and filled in as an amazing wellspring of carbon [65]. A gigantic measure of waste produced by enterprises can be refined and utilized as phenomenal elective substrates. Thus, producing low cost biosurfactants through this approach is an alternative for production of biosurfactants as compared to that of petroleum derived methods.

## 4 Properties

Biosurfactants have certain unique properties that differentiate them from chemical surfactants and make them a preferable choice for utilization in various formulation developments and aggregation studies. Some of the important properties of biosurfactants have been discussed in this section explaining their advantages over the common chemical surfactants.

### 4.1 Excellent surface and interface activity

In terms of their surface and interfacial activity, biosurfactants have been found to be more effective and efficient than chemical surfactants. Furthermore, it has been noted that their CMC values are significantly lower than those of chemical surfactants [66]. Due to these properties, surface tension of a liquid surface can be decreased by using much less amount of biosurfactants. According to the findings of Cooper *et al.*, the biosurfactant surfactin generated by *B. subtilis* effectively lowers the interfacial tension of water and hexadecane to less than 1 mN m^−1^ and reduces the surface tension of water to 25 mN m^−1^ [67]. According to a different study, *Pseudomonas aeruginosa*-produced rhamnolipids lower water’s surface tension to 26 mN m^−1^ and the interfacial tension between water and the hexadecane solvent system to less than 1 mN m^−1^ [68].

### 4.2 Biodegradability

Biosurfactants are environmental friendly compounds and can easily be degraded into simpler metabolites when compared to the chemical surfactants. The biosurfactants derived from marine micro-organisms have been effectively used in various bioremediation and biosorption processes [69]. They have been reported for biosorption of cochlodinium having high removal efficiency of more than 90% within half an hour. Apart from this, the biosurfactants have also been used for biosorption of phenanthrene and polycyclic aromatic hydrocarbons from aquatic surfaces [68, 69].

### 4.3 Anti-adhesiveness

The biosurfactants are considered as more appropriate anti-adhesive agents to alter the hydrophobicity which helps in decreasing the adhesion of microbial organisms to the surface of a biofilm [70]. This property of biosurfactants helps in formulating a stable biofilm as foreign sources such as micro-organisms, hydrophobicity or electrical charges do not adhere to the surface of biofilm. Some examples of this property include the surfactant produced from *Streptococcus thermophilus*, that did not allowed the accumulation of other thermophilic strains over a steel surface and prevented it from rusting [71]. In a similar study, a biosurfactant produced from *P. fluorescens* did not allowed the adhesion of *Listeria monocytogens* on the surface of a steel [72].

### 4.4 Emulsifying agents

Biosurfactants are better emulsifying agents than chemical surfactants as they do not degrade easily and forms stable emulsions that can be stored for a long time [73]. Cirigliano and Carman demonstrated this property by preparing the emulsions from liposan, produced from *Candida lipolytica* and compared it with the chemical based surfactant emulsions. The results suggested better emulsifying property of the biosurfactant with better stability [52].

### 4.5 Low toxicity

The biosurfactants are considered as very low to non-toxic compounds for use in pharmaceutical, cosmetics and food industries [74]. Since the high concentration of chemical surfactants in consumable products can lead to various side-effects and toxic reactions, the biosurfactants generally metabolize to form non-toxic products and thus widely used in these sectors. The comparative toxicity studies of chemical and bio-surfactants were performed by Poremba *et al.* The research work reported that rhamnolipids were 10 times less toxic than the chemical surfactant (Corexit) used in the study [75].

### 4.6 Thermophilic

The temperature and pH tolerance capacity of biosurfactants makes them a preferable choice over chemical surfactants. Biosurfactants are known to resist the changes in temperature and pH to a wide range. McInerney *et al.* reported the temperature resistant capacity of lichenysin, produced from *Bacillus licheniformus* up to 50 °C and pH resistance over a range of 4.5–9 [76]. Another study reported that the production of biosurfactant from *Arthrobacter protophormiae* was temperature as well as pH resistant between the range of 30 ^°^C–100 °C and 2–12, respectively [77].

## 5 Characterization

The biosurfactants can be characterized using various spectroscopic techniques. From the Nocardia sp. L417 strain, Kim *et al.* developed two different types of biosurfactants and characterized them using techniques such as ammonium sulphate fractionation, chilled acetone and hexane treatments, silica-gel column chromatography, and Sephadex LH-20 gel filtering [78]. In an another study, Kruijt *et al.* performed the purification and chemical analysis of biosurfactants developed from *Pseudomonas putida* 267 strain by using reverse phase high performance liquid chromatography (RP-HPLC) and liquid chromatography-mass spectrometry (LC-MS) techniques [79]. Similarly, Janek *et al.* utilized ultra performance liquid chromatography-tandem mass spectrometer (UPLC-MS) method to identify the structures of novel biosurfactants produced from *Pseudomonas putida* BD2 isolate of Arctic bacterium [80].

In a recent research, *Ribeiro et al.* evaluated the cytotoxic activity of a biosurfactant derived from *Candida* *utilis* using MTT assay (3-(4,5-dimethylthiazol-2-yl)-2,5-diphenyltetrazolium bromide). This test was created by utilising mouse macrophage and fibroblast cells, which were maintained at 37 °C in a culture flask with 5% CO_2_ [81]. Another interesting work by Yang *et al.* describes the utilization of biosurfactant produced from *B. subtilis* Y9 as insecticidal metabolite. The biosurfactant property was identified by performing column chromatography and preparative high performance liquid chromatography (HPLC) analysis [82]. Moro *et al.* isolated a new surfactin from oil-contaminated soil by employing qualitative drop-collapse test, oil-spreading and emulsification assays. The resulting biosurfactant thus obtained along with other isolates of *B. subtilis* were characterized by using thin layer chromatography (TLC) and ultra-high-performance liquid chromatography-high-resolution mass spectrometry (UHPLC-HRMS) techniques [83]. Similar characterization methods were used by Luong *et al.* for purification of trehalolipid biosurfactants obtained from *Rhodococcus erythropolis* S67 strain. The study was performed at low temperatures (10 °C and 26 °C) and the resulting biosurfactants were identified using mass spectrometric (MS) analysis [46]. In the latest published research work, Rosas-Galván *et al.* evaluated the effect of carbon and nitrogen on the biosurfactants produced from *Serratia marcescens* and its isogenic SMRG-5 strains. The molecular structure of biosurfactants was further identified by gas chromatography-mass spectrometry (GC–MS) analysis [84].

Microbial surfactants can be sorted by their method of activity, sub-atomic weight and general physicochemical properties. They are generally classified according to their synthetic strategy and sub-atomic weight, as low (for example glycolipids and lipopeptides) and high atomic weight (for example polysaccharides, proteins and lipoproteins) surfactants. Microbial surfactants create an atomic interfacial coating in heterogeneous frameworks that affects the initial surface’s wettability and surface vitality. Other than bringing down the fluids surface pressure, these can incredibly affect the interfacial conductance and mass exchange, as they likewise bring down the interfacial strain between diverse fluid stages on the interfacial limit existing between immiscible stages.

## 6 Applications

Chemical approaches are used to obtain commercially viable biosurfactants from the petrochemical sector. Microbes and other sources have been investigated for the manufacture of surfactants in an effort to limit the use of chemicals. Examples of organic surfactants include saponins produced by plants, biliary salts produced by people, and glycolipids secreted by microbes [85, 86, 87]. Since the term is being used broadly, it also includes some of the by-products made by yeasts, bacteria, and some filamentous bacteria, such as emulsifiers and dispersion agents, which are frequently utilised in the petrochemical industry, food industry, and pharmaceutical industry. Surfactants are frequently utilised in the detergent and soap-making sectors of the cleaning industry. Surfactants are also employed in increased oil recovery and bioremediation of hydrocarbon contamination [88]. In the food industry, biosurfactants are also employed as emulsifiers. Some major applications are:

### 6.1 Gene transfection

Transfecting genes into a cell are the core of clinical quality treatment. Lipofection carried out by cationic liposomes is seen as a promising strategy for imparting external qualities into the cells, as liposomes have high transfection productivity, less toxicity, simple arrangement and are application focused [89, 90]. The effectiveness of transfection is influenced by the physicochemical characteristics of cationic liposomes, such as lipid pressing density, zeta potential, and shape. By starting the layer integration between the target cells and the cationic liposomes, the biosurfactant increases the effectiveness of transfection.

A recent research by Madihalli *et al.* discovered that monolayers made of L-α-dipalmitoyl phosphatidylcholine (DPPC) containing MEL-A had more prominent transfection effect than those containing just DPPC. Mannosylerythritol lipids (MELs) are structurally classified into four different types i.e. MEL-A, MEL-B, MEL-C and MEL-D. MELs have a property of self-assembly that can be used in chain delivery and drug encapsulation processes [91]. Additionally, as the continuing environmental, economic, and healthcare issues throughout the world continue to worsen, biosurfactants provide hope. With this line of reasoning, research is currently focusing on developing viral vectors and investigating non-viral vectors for DNA editing [92].

### 6.2 As drug delivery agents

Biosurfactants are widely used as drug delivery and therapeutic agents in pharmaceutical industries. The study performed by Baferani *et al.* shows that biosurfactants exhibit properties like emulsification, frothing, detergency and scattering [93]. Lecithin can be used to blend rhamnolipids and sophorolipids to create biocompatible microemulsions. Because of this, their phase behaviour is resistant to temperature and salt changes, making them ideal for use in drug administration [94, 95]. A recent work by Sadeghi *et al*. also confirmed that microemulsions obtained by utilizing biosurfactants are thermodynamically steady and its isotropic frameworks generate spontaneously. Apart from their high solubilization limit, ease of preparation, and long-term stability, biosurfactants-aided microemulsions are thought to be particularly encouraging fluid vehicles for future drug delivery systems [96]. In addition, compared to conventional drug administration methods, the merging of an emulsion into a gel boosts its strength and effectiveness. Souto *et al.* proposed the semi-solid hydrogels for drug delivery system, for the benefit of environmental bioremediation in biotechnology. As an alternative to pesticides, these delivery methods give producers a platform to save production costs and their environmental impact [97].

### 6.3 In preparation of micro-emulsion systems and nano-particles

A watery stage, oil-stage, a surfactant, and a co-surfactant are required to build a microemulsion-based colloidal drug delivery system, according to a study by Feng *et al.* [98]. Potentially isolating the dispersed phase from the continuous phase, these structures can solubilize the hydrophobic/hydrophilic medicines inside the dispersed phase’s structural core. These structures exhibit increased solubilization and very low interfacial tension at oil and water, making them appropriate for application in the delivery of medications. They are also thermodynamically stable. In a different work, Nguyen et al. created an alcohol-free micro-emulsion system using rhamnolipid biosurfactant and its combinations. Phase diagrams, which provide significant information on the selection of surfactant systems and their ratio for future studies, were used to effectively illustrate the results [99]. Another study that involves creating lecithin-based micro-emulsions from rhamnolipid and sophorolipid was conducted concurrently with this one [100].

Size and shape of nanoparticles are used to describe them. The current processes result in a lot of waste and inadequate nanoparticles with limited functionality. Microbial surfactants are being employed as alternatives to follow greener bio-handling. Gold nanoparticles are mostly used in medicine, quality delivery, focused therapy, and imaging advancements [101, 102, 103]. Since biosurfactants efficiently create liquid crystals in aqueous solutions, they can be used to produce superior nanomaterials. In addition to silver and iron oxide nanomaterials, biosurfactants assisted gold nanoparticles have also found considerable use in medical and health technologies [104, 105]. Additionally, Aminabhavi *et al.* used ultrasonic irradiation to create the nanostructured materials using green sonochemical methods. The development in the production of tailored nanomaterials with diverse dimensionalities that are safe for the environment has been described [106]. Verma and Das used water-in-oil and oil-in-water micro-emulsion methodologies to explain the impacts of nanomaterials [107]. In addition, it has been discovered that biosurfactants and nanoparticles can be combined with synthetically produced surfactants to produce brand-new, more effective surface active agents for better oil recovery. Based on research in the literature, it was discovered that these formulations can improve the flow back of the injected stimulation fluids and further mobilise the oil to be extracted from the matrix and micro-fractures [108].

### 6.4 Biofilm disruption

Bacteria have the capacity to modify how they express themselves in response to their surroundings. Quorum sensing (QS), a rare phenomena that only some gram positive (+ve) and gram negative (−ve) bacteria have, allows them to interact with one another and detect their own cell density [109]. Although it also depends on the nutritional environment that is available for bacteria to carry out QS activity, this ability of bacteria has been linked in the creation of biofilms. The cells connected to the surface begin to organise into clusters of micro-colonies that are surrounded by other cells known as water channels during the biofilm development and maturation event [110, 111]. In the food industry, biofilms are a major source of disease and contamination. Therefore, the formation of biofilm on food surfaces is avoided by using biosurfactants to disrupt the adherence of microbial cells to the surface, thereby preventing food deterioration. According to Huang *et al’s* illustration, the application of rhamnolipids and surfactin in particular reduced the adherence of microbial cells to polypropylene surfaces [112]. In order to stop the development of biofilm in the oral cavity, biosurfactants can also be found in health and hygiene products.

**Figure 2:**
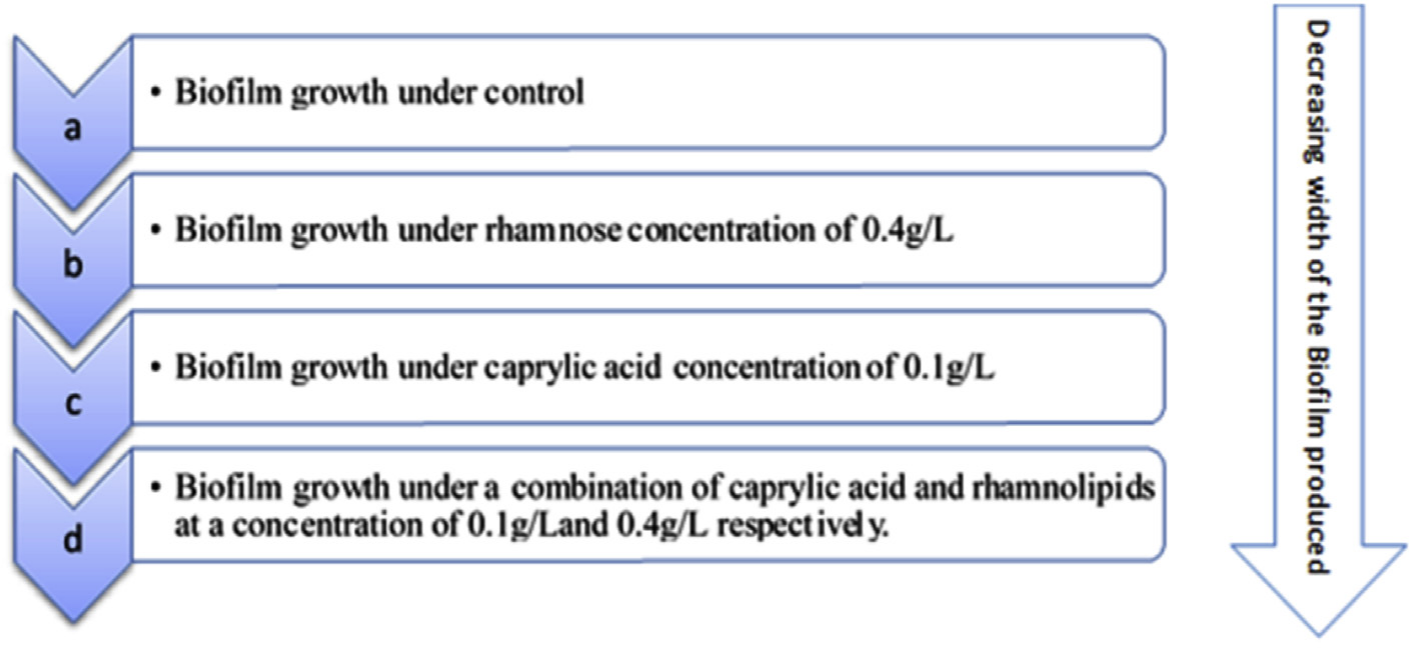
Biofilm formation in the presence of different biosurfactants.

Microbial surfactants, often known as biosurfactants, have the capacity to degrade biofilms and inhibit adhesion. Enzymatic surfactants like lauryl-glucose, which are created from synthetic materials, destroy the biofilms that many bacteria and fungus produce [113]. Rhamnolipids, a group of surfactants generated from *Pseudomonas aeruginosa*, are effectively employed in biofilm removal. By causing cellular clustering and subsequent colony formation on the already-existing biofilms, the overproduction of rhamnolipids prevents the formation of biofilms [114, 115]. Cells from biofilms are also dispersed by them. *Pseudomonas aeruginosa* biofilms were developed on flow cells in a recent study by Rienzo *et al.* Using a peristaltic pump, oxygen and nutrients were delivered to the chamber from a flask containing 50% tryptic soy broth. The used medium was collected in a waste container. To check the development of a biofilm, the cells were labelled with LIVE/DEAD cells. Fluorescence microscopy was used to observe the biofilm that was generated on cover slips to ascertain the impact of various treatments. Rhamnolipids were found to limit the bacterial biofilm production, as evidenced by a decrease in the number of active cells and the formation of reddish-brown luminous cells (Figure 2). Adjuvant addition, such as caprylic acid, had no impact on biofilm dispersal on its own. Rhamnolipid and caprylic acid together had a stronger impact on biofilm deterioration. Moreover, even at low doses, this combination proved extremely efficient [49].

Antibiotics and biocidal substances are highly resistant to biofilms, which are heterogeneous formations. Rhamnolipids have been linked to improving bacterial transport across columns by causing steric hindrance as a result of the bacteria’s contact with the surface. Although rhamnolipids have been evaluated with a variety of strains and conditions, they consistently appear to impair cell-to-cell adhesion and biofilm disruption [116]. When rhamnolipid and caprylic acid are used together, biofilm disruption is found to be more effective [117]. This behaviour may have emerged as a result of a synergistic impact. Rhamnolipids appear to eliminate microcolonies and extracellular polymeric substances, changing the biofilm environment and making cells more vulnerable to the effects of caprylic acid, which permeates cell membranes and causes cell death. Additionally, mechanical disturbance caused by shear forces may result in the dispersal of biofilm [118, 119]. The detailed discussion of implants and the illnesses connected to them, which are brought on by the presence of foreign material during biofilm development, followed. Current research has been altered by the development of novel techniques and treatments to inhibit bacterial growth or division that results in cell death or dormancy, cause biofilm breakup, or look into ways to prevent biofilm formation due to the rise in antimicrobial resistance [120].

### 6.5 As antioxidants

Certain biosurfactants are known to possess antioxidant properties which have been utilized in pharmaceutical formulation process. Mannosyl erythritol lipids (MELs) are versatile biosurfactants having interfacial and biochemical properties. MEL-C possesses free radical ion scavenging activity and has the highest antioxidant activity [121]. Therefore, it provides the highest protection and is actively used in skincare cosmetics manufacturing. Similar activity has been shown by RW1, a biosurfactant derived from *Bacillus subtilis,* proving a natural alternative to antioxidants [122].

### 6.6 As anti-adhesives

Numerous biosurfactants display antimicrobial properties, as well as anti-adhesive properties. Rhamnolipids and surfactin possess anti-adhesive properties against *Listeria monocytogenes* and *Staphylococcus aureus* on polystyrene surfaces [123, 124]. Similar anti-adhesive activities were shown by biosurfactants produced from *Candida sphaerica* against many strains of *Streptococcus*, *Staphylococcus*, and *Candida* on plastic tissues [125]. These properties of biosurfactants promote their use as coating agents or in cooking utensils to reduce fouling, as well as in dairy industries. Rodrigues *et al.* depicted the utilization of biosurfactants as probiotics like *Lactobacillus acidophilus* strain [126].

### 6.7 In food industries

In a quest to find natural sources for obtaining biosurfactants rather than those obtained from genetically modified plants, many natural resources, mostly microbial sources are being explored as they are inexhaustible. Emulsions are widely used in food industries and they contain at least one non-soluble liquid mixed in another one in droplet form. Their stability can be improved by using biosurfactants [127]. Emulsification imparts texture, consistency and dispersion patterns to food products. An emulsifier stabilizes the emulsion by managing the mixture by aggregation of globules [128]. Additives are compounds having zero nutritional value, but are added to improve chemical and biological properties during manufacturing, packing and transport. Additives provide properties like thickening, gelling, stabilizing and emulsifying. Glyceryl monostearate, a synthetic additive is widely used in food industries [15].

Emulsifiers are typically surface-active substances that helps in producing emulsions by lowering interfacial tension and improving their stability. Low molecular weight amphiphiles are used to provide stability to various food products as beverages, dressings, sauces etc. [129]. Emulsifiers like lecithin and gum Arabic derived from plants are also being used but they suffer some functional limitations. Lecithin is employed for production of cocoa powder which are a common ingredient for many desserts [130]. Many of the modern-day food products like mayonnaise, butter, cream, salad dressings are produced using emulsions. Therefore, natural resources are being explored to produce bio-emulsifiers. Manno-proteins produced via *Saccharomyces cerevisiae* stabilizes water-oil emulsion and is used to produce various food products [131]. The majority of biosurfactants made by yeasts and bacteria can function in a variety of pH and temperature conditions, hence they are employed in place of traditional emulsifiers. Microbial surfactants are also used to provide texture, to improve shelf life and to modify rheological properties of dough. They are also used to enhance texture, delay staling and stabilize fats in ice creams [132].

**Figure 3:**
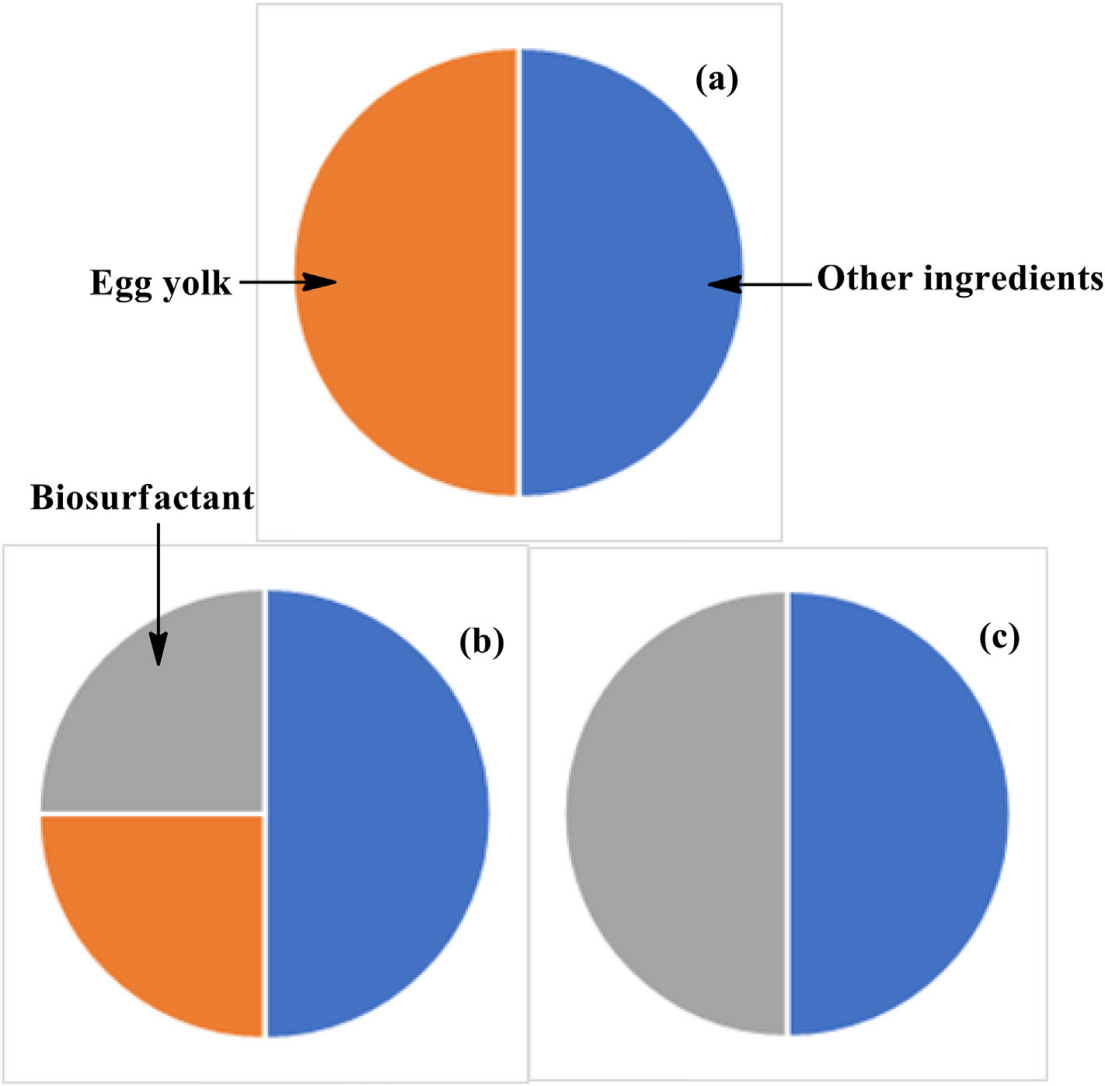
Representation of formulations used in cookies: (a). Standard Formulation (b). Formulation having 50% egg yolk replaced with biosurfactant (c). Formulation having 100% egg yolk replaced with biosurfactant.

Microbial biosurfactants are also utilized for making cookie formulations. In a latest research work, Ribeiro *et al.* prepared dough for the cookie in 3 different ways as represented in Figure 3. The standard dough had ingredients used in normal formulations, while in other formulation, 50% of the pasteurized egg yolk used in standard formulations was replaced with biosurfactant UFPEDA1009 produced by *Candida utilis*. In the last formulation, the pasteurized egg yolk was fully substituted with the microbial biosurfactant, and they were ultimately baked. After baking, physical characteristics such as shape, texture, diameter, thickness and spreadability of the cookie were characterized. Texture analyser was used to record the texture profile of the cookie dough before baking, while only firmness could be recorded after baking them. It was found that the protein content in both the alternative formulations was lower as compared to that of standard formulation. Since the biosurfactant used in this study was a carboxylic acid derivative from *Candida utilis*, thus it lacked proteins. However, the formulation which replaced egg yolk by 100% had high lipid contents, which happens to be the reason for high energy content of this formulation [81].

### 6.8 Biodegradation

Surfactants have the tendency to make less soluble and recalcitrant chemicals more accessible to microorganisms during the biodegradation process. Compounds being less soluble i.e. with high hydrophobicity get bonded strongly to organic matrix and are not desorbed easily to be available for degradation. During the hydrocarbon degradation, low molecular weight hydrocarbons are degraded first leaving recalcitrant compounds [133]. Since surfactants increase the solubility in water and lead to better mass transfer, they are used to enhance biodegradation of hydrocarbons. Utilizing this application of biosurfactants to facilitate diesel hydrocarbon biodegradation, Franzetti *et al.* selected a suitable biosurfactant by assessing its physico-chemical properties, biodegradability and its effect on the rate of biodegradation. For the selection purposes, environmental fate and toxicity were assessed by using K_OC_ and EC_50_ using a QSAR model. K_OC_ is defined as the organic carbon-water partition coefficient in the ratio of chemical mass adsorbed on the soil per unit mass of the organic carbon of the soil split by the equilibrium concentration of chemical in aqueous solution [134]. EC_50_ (half maximal effective concentration) is defined as the concentration of a poisonous compound which induces a response halfway in between the maximum and baseline value, and is used to determine toxicity [135]. QSAR is Quantitative Structure Activity Relationship, a procedure using which the chemical structure of a molecule is corresponded with a definite property like biological activity etc. [136]. Respirometric analysis was used to assess the biodegradability of the surfactants, using Biochemical Oxygen Demand (BOD) apparatus. Solid phase respirometric tests were conducted to obtain biodegradation of surfactants in soil samples. The studies concluded that mobilities along with the amount of time the surfactant remains in the soil is affected by biodegradability and sorption characteristics [137]. For being efficient in biodegradation, the surfactant must be less toxic, persistent and must not have affinity towards soil. Non-ionic surfactants are more biodegradable and less toxic as compared to others [138, 139].

### 6.9 Bioremediation

The intricate physical-mixture interactions at interfaces serve as a representation of the degree of interaction between natural and inorganic poisons. Toxins bind to the soil’s surface and then sequester themselves there, generating a liquid known as the Non-Aqueous Phase Liquid (NAPL). Natural substances known as NAPL are long-lasting pollutants that are insoluble in water. Continuously replacing dissipated concentrations with them reduces the amount of contaminated particles that can be biodegraded in the water phase [140]. Enhancing the breakdown of biological substances to lessen the impact of poisons is known as bioremediation. The practise of bio-augmentation involves introducing cultured bacteria to promote pollutant degradation, reinforced by the use of surfactants to promote pollutant desorption, which enhances the decomposition of hydrocarbons [141]. Surfactants are used in oil washing and oil pipe cleaning to recover secondary oil. For desorption purposes, a variety of biosurfactants and synthetic surfactants are used. Due to their expensive manufacturing costs, biosurfactants are not frequently employed in bioremediation. This issue might be remedied by employing less expensive substrates, like agro-industrial waste and industrial oil waste, to cultivate the microorganisms that manufacture these microbial surfactants. The type of biosurfactant generated is significantly influenced by the quality and quantity of nitrogen and carbon present in the substrate and soil. Because there is less nitrogen available, more biosurfactants are produced, which helps with bioremediation [142, 143]. In order to increase the microbial activity that results in higher synthesis of biosurfactants, nitrogen is frequently omitted from the supply of bioremediation systems.

The ability to make biosurfactants and to separate hydrocarbon-degrading bacteria from oil-contaminated areas is made possible by the addition of naturally hydrophobic materials to the culture medium [144]. For this purpose, sources of carbon include aliphatic and aromatic hydrocarbons. The main contributors to soil pollution are organic compounds and metallic contaminants. Poly-cyclic aromatic hydrocarbons (PAH) and poly-chlorinated biphenyls (PCB) are examples of organic hydrocarbons, with PHA being more prevalent as a result of their use in the refining of crude oil [145]. The non-linear rate-limited biodegradation and sorption processes in soil are what determine how contaminants are transported there. The majority of hydrophobic pollutants attach by sorption to organic soil components. Humic acid and non-humic substances (such as proteins and waxes) are both engaged in the sorption process [146].

Pollutant degradation in soil is a result of physico-chemical and biological interactions between the pollutants and other biological elements. Numerous parameters, including the availability of pollutants to the microbes, pH, water, aeration, nutrients, temperature, etc., regulate degradation through microbial activity. A thorough understanding of the microorganism’s metabolic processes might improve biodegradation [147]. As a result of greater oil breakdown caused by the formation of a stable oil-in-water emulsion by microbes, the nutrients available to the bacteria are increased. In natural systems, this phenomenon is produced by using the autochthonous surfactant bio-population, which results in the development of stable emulsions [148]. Additionally, microbes have the capacity to attach to and create complexes with the contaminants, which makes the process of degrading pollutants more laborious and challenging. As a result, by alkylating heavy metals, bacteria can eliminate them [149]. Microbial surfactants are typically utilised to degrade oil spills because synthetic surfactants are hazardous to the microorganisms that break them down. Composting systems are employed in this procedure to improve oxygen transport and, as a result, decrease soil pollution within a few months [150]. Introducing bacteria with the ability to break down hydrocarbons with high oxygenase activity, like *Acinetobacter*, is a systematic technique to promote the breakdown of crude oil in contaminated environments. Additionally, several complexes made from biosurfactants are separated using the solvent methyl tertiary-butyl ether [151].

One of the reasons that organic and metallic pollutants stay in soil for such a long time without being destroyed is because the contaminants are not available to the micro-organisms for breakdown. Furthermore, the interaction of the contaminants with the environment and the microorganism may not be adequate, preventing the microorganism from performing the necessary catabolic activities. Therefore, it is crucial to produce biosurfactants from bacteria and fungi since they have a propensity to solubilize hydrophobic pollutants, allowing for their simple destruction. Amato *et al.* highlighted the benefits and drawbacks of various biotechnological strategies in their discussion of multiple patents on the subject of bioremediation of sediments contaminated with polycyclic aromatic hydrocarbons. This knowledge may be useful in resolving problems and enhancing the efficiency of bioremediation. It can serve as a benchmark of data for bioremediation firms to find and investigate the most promising ways that are suitable to the field [152].

### 6.10 Agriculture

The health and productivity of plant crops are monitored on a regular basis to ensure the quality of the food and its availability to meet the increasing demands of growing population. Metabolic compounds referred to as green/renewable surfactants are widely used nowadays to improve the quality and quantity of crops. Epiphytic and endophytic microbes are involved in biosurfactant production that aids in seed germination [153]. They also inhibit the action of phytopathogens and helps in bioremediation by acting as biocontrol agents. Since the use of pesticides on plants must be avoided to obtain chemically free plant products; rhizospheric bacteria have been explored by researchers that secrete metabolites which increase the yield of plant products [154, 155]*.*

*Bacillus* and *Pseudomonas* are the majority of microbes in Rhizobacteria group, which secrete metabolites having versatile applications as plant growth promoters, fungicidal agents like siderophores, phenazines, pyrrolnitrin and cyclic peptides. The enzymes like chitinase and glucanase also possess hydrolysing activities [156]. These green compounds possess lower critical micellar concentration compared to that of synthetic ones and lead to higher decrease in surface tension of oil/water or air/water interfaces to a minimal value. Rhamnolipids and lipopeptides are the majorly studied biosurfactants, which have tremendous applications in agricultural field, along with other industries like pharmaceuticals, cosmetics, petrochemicals, food etc. They are well known to promote seed germination, reduce the growth of phytopathogens and induce resistance in the plants [157].

**Figure 4:**
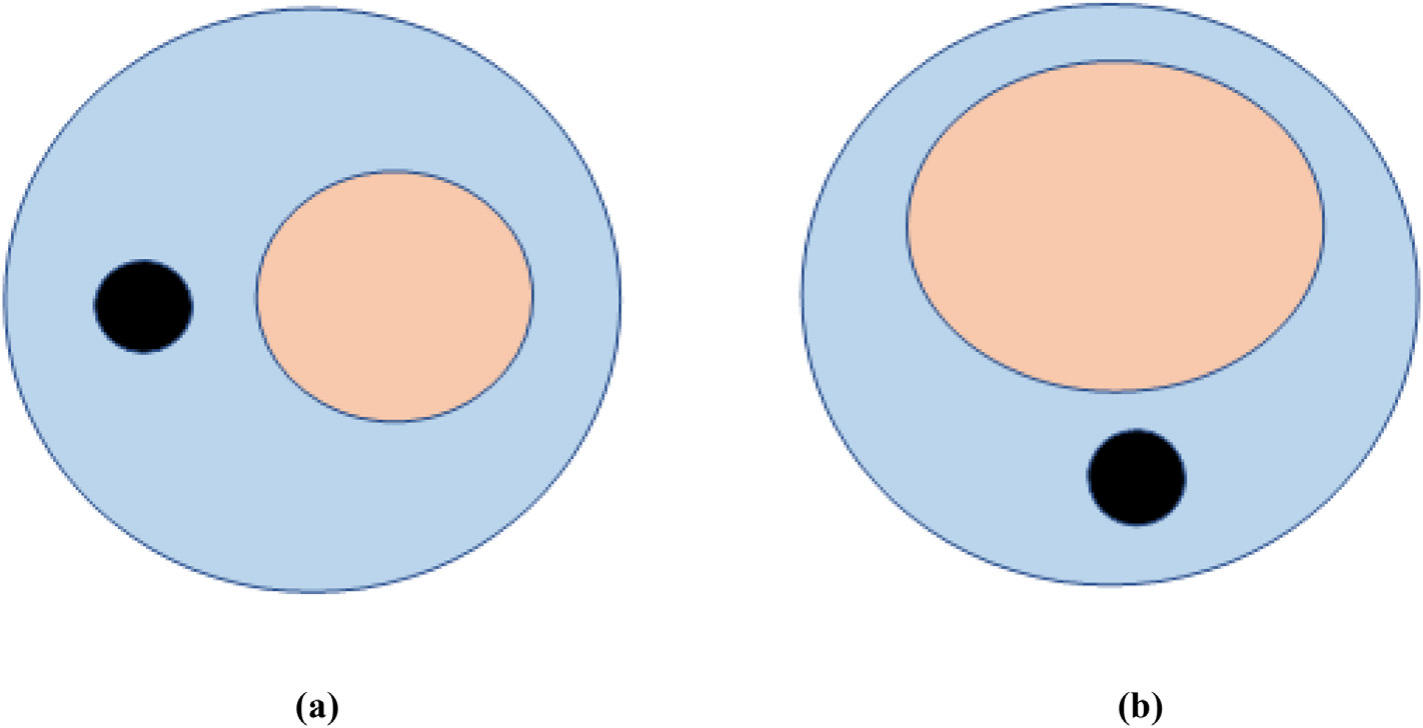
Antifungal activity of the biosurfactant against the chosen fungi representing: (a) *Fusarium oxysporumf*. sp. *ricini* inhibited by means of lipopeptide. (b) *Fusarium oxysporum f.* sp. *ricini* inhibited by means of rhamnolipids.

In a recent study, *Bee* et al. utilized the rhizosphere portion of many cereals for bacterial isolation. The isolated bacteria were tested for ammonia production, antifungal activity, phosphates solubilising ability and capability to produce indole acetic acid. The antifungal ability of bacteria was evaluated against *Fusarium oxysporum f.* sp. *ricini* which was ranged between 72 and 80%. From the results, it was observed that 67% of *Pseudomonas* sp. and 58% of *Bacillus* sp. possessed PGP traits as mentioned in Table 2. Further, it was concluded from the study that raw biosurfactants containing rhamnolipid and lipopeptides showed fungicidal activity against *Fusarium oxysporum f. ricini.* sp. as represented in Figure 4.

**Table 2:** List of various traits tested and reagents used by *Bee* et al.

S. No.	Trait tested	Reagent/Protocol used
1	Ammonia Production	Nessler’s reagent
2	Solubilization of Phosphate	Pikovskaya’s Agar having tricalcium phosphate
3	Phytase activity	Phytic acid medium
4	Production of siderophore	CAS agar plate system
5	Production of IAA	Nutrient agar supplemented with L-tryptophan.

Rhizobacteria have been known to promote plant growth activity, alongside which they also depict strong antifungal or fungicidal activities making them a preferable choice as bio-inoculants and in agriculture. Crop yield and sustainable agricultural conditions still prevail due to the regular application of chemicals which also pollute the environment. To overcome this shortcoming, non-toxic bacterial metabolites which tend to inhibit the growth of fungal pathogens and enhance seed germination are suitable alternatives for chemical compounds [158]. In a recent research, Silva et al. presented many methods for formulating simultaneous production and process optimization using statistical and genetic tools in relation to process parameters and variables. The primary obstacles to overcoming the large-scale manufacturing of fungal biosurfactants have been identified to be economic concerns and low process productivity. The economic viability of using waste streams for biosurfactant synthesis can only be achieved with careful analysis between cost reduction and low yield due to the creation of inhibitory chemicals [159].

## 7 Conclusion

Biosurfactants have certain unique properties like excellent surface and interface activity, biodegradability, anti-adhesiveness, emulsifying properties, low toxicity and thermal resistance. Due to this diversity in properties they are used in a wide array of industries like petrochemical, petroleum, coal, metallurgical and mining industries, dairy, cosmetics, pharmaceuticals, food, agricultural and fertilizer, cosmetics, food and beverages etc. These compounds have been majorly obtained from bacteria and this is well documented. The bio-surfactants are also obtained from fungi, though there is less data on this subject. Many tropical species of plants worldwide have been used to isolate numerous endophytic microorganisms which produce biosurfactants. They are currently not used at industrial level in a large scale, since their effect needs to be first determined by an experimental setup in greenhouse conditions and actual field studies, following which it can be stated if they can act as potential alternatives against the chemical agents. This review article emphasizes on the advantages of using biosurfactants and discusses various sectors where they have been successfully utilized and resulted in environmental friendly and better products than chemical surfactants.

## 8 Future perspective and challenges

Microbial derived surfactant molecules are generally non-ionic at low pH. They are anionic at high pH because of nearness of carboxylic gatherings. Microbial surfactants can possibly be utilized for the targeted-drug delivery. Additionally, pH-delicate liposomes containing rhamnolipids and di-oleoyl-phosphatidyl-ethanolamide have been depicted as effective frameworks for cytoplasmic conveyance of particles into cells. There is a critical need to investigate microbial frameworks to acquire surfactants and use them as effective drug delivery systems. Industries are seeking to find natural alternatives for biosurfactant production for their utilization at industrial scale. Since biosurfactants are economically a bit costly to prepare in current scenario, it is essential to find ways to decrease the cost of production. Through the application of specialized cost-effective testing and discussion, it might be possible to find a breakthrough to allow the use of biosurfactant at a large scale. Some latest research works have shown promising results in developing bacterial culture using poultry waste flour and buttermilk, waste cassava fry oil, agro-industrial waste etc., thus serving as low cost alternative for producing the biosurfactants. Also, *Piper hispidum* has also been identified as a potential alternative for the isolation of micro-organisms producing biosurfactants, which can be utilized in various industries. Biosurfactants owing to their higher biodegradability, biocompatibility and lower toxicity can be optimized by tweaking fermentation conditions so as to obtain biosurfactants at an affordable cost.

## Declarations

### Author contribution statement

All authors listed have significantly contributed to the development and the writing of this article.

### Funding statement

This research did not receive any specific grant from funding agencies in the public, commercial, or not-for-profit sectors.

### Data availability statement

No data was used for the research described in the article.

### Declaration of interests statement

The authors declare no conflict of interest.

### Additional information

No additional information is available for this paper.
